# Revealing hidden fall accident patterns in Indian construction using association rule mining

**DOI:** 10.3389/fpubh.2026.1861573

**Published:** 2026-08-05

**Authors:** Vigneshkumar Chellappa, Ahmed Jalil Al-Bayati, Jozef Selín, Alžbeta Suhányiová

**Affiliations:** 1 School of Design, The Hong Kong Polytechnic University, Kowloon, Hong Kong SAR, China; 2 Department of Civil and Architectural Engineering, Lawrence Technological University, Southfield, MI, United States; 3 Faculty of Civil Engineering, Technical University of Kosice, Kosice, Slovakia; 4 Faculty of Mechanical Engineering, Technical University of Kosice, Kosice, Slovakia

**Keywords:** construction industry, fall accidents, construction accidents, occupational health and safety, workers’ health & safety, rule mining, fall from height

## Abstract

**Background:**

Falls from height are a leading cause of fatalities worldwide, yet the lack of systematic accident data has limited the development of targeted prevention strategies in India. This study aimed to identify patterns and causal factors associated with fatal fall accidents in the Indian construction industry between January 1, 2019, and March 31, 2025, using association rule mining.

**Methods:**

Owing to the unavailability of official government accident records, 126 fatal fall cases were collected from three major national newspapers. The cases were analyzed using the Apriori algorithm to identify frequent associations among accident-related variables. Strong association rules were evaluated across six consequence categories: causal factors, workers’ state of origin, location of fall, occupation, state/region, and age.

**Results:**

The analysis generated 148 strong association rules. The absence of fall protection emerged as the dominant causal factor, consistently observed across most accident scenarios. Workers aged 18–25 years, general laborers, and migrant workers from Uttar Pradesh and Bihar were disproportionately represented among fatal fall victims. Scaffold-related and structure-related falls were the most common accident locations and were strongly associated with missing or improperly used fall protection. Maharashtra, Delhi, and Haryana were identified as the highest-risk regions.

**Conclusion:**

The findings show that even in data-scarce environments, systematic analysis of publicly available information can provide actionable safety insights. Fall prevention efforts in India should prioritize the provision and proper use of fall protection equipment, focus on young migrant workers and general laborers, strengthen enforcement in high-risk states, and establish a mandatory national accident reporting system to support evidence-based safety policy.

## 1 Introduction

The construction industry plays a vital role in driving economic growth, contributing significantly to the Gross Domestic Product (GDP) of many countries (1). The industry also employs about 7% of the world’s workforce, making it a crucial sector for job creation (2). Despite its importance, the construction industry has long been recognized as one of the most hazardous industries globally, posing significant risks of severe injuries and fatalities. These high accident rates can be attributed to a complex interplay of factors, including organizational culture, industry fragmentation, and the dynamic nature of worksites (3, 4). According to OSHA, the four major hazards that contribute to construction accidents are: falls, being struck by objects, electrocution, and being caught in/between (5). Among these, falls account for a large share of construction-related fatalities and injuries worldwide, making them a significant contributor to such outcomes (6–8). For example, in Korea, falls accounted for 59.8% of all construction accidents, according to the Ministry of Employment and Labor in January 2023. Similarly, in 2019, 415 fall-related accidents occurred, accounting for 53.69% of all accidents (9, 10). In Hong Kong, construction falls accounted for 49% of all fatalities (70). Similar trends have been reported in multiple regions, including the USA, Malaysia, and Turkey (6, 8, 11, 12) with research indicating that construction falls are a leading cause of fatalities in these areas. These elevated accident rates can result in project delays, work disruptions, and significant financial losses for all parties involved (4, 13, 14).

Many studies have investigated trends and patterns of falls in the construction industry. For instance, the OHSA accident database and NIOSH fatal incident reports were used to examine the fall accident trends in the USA construction industry (15–17). Similarly, Lin et al. (18) analyzed the Taiwan Council of Labor Affairs (CLA) database to investigate accident patterns. Besides, numerous studies have examined global trends in fatal falls within the construction industry (3, 4, 7, 19–24). This analysis identified several key factors contributing to falls, including the ages of the workers involved, the locations of incidents, and the types of projects most affected (16, 17, 25). Numerous studies have linked falls to several contributing factors, including a lack of safety guidance, inadequate worksite supervision, insufficient protective barriers, unsecured floor openings, and improper use of fall-protection equipment (4, 8, 26). These studies inform the prioritization of fall-prevention strategies and guide practitioners and stakeholders in implementing effective fall-reduction measures in the construction industry. For example, identifying potential fall locations using Building Information Modeling [BIM; (27)]; a significant source of construction falls (28); and developing targeted safety training programs for young workers (29), who are disproportionately involved in fall incidents (14, 15, 18). These studies demonstrate that identifying patterns of fall incidents is crucial for developing targeted prevention strategies and improving overall fall safety in construction.

Despite its significance, research on fall trends in the construction industry, particularly in developing regions like India, remains limited. As the nation’s largest employer, India’s construction sector provides livelihoods for approximately 31 million people (30, 31). However, the industry has a history of weak enforcement of workplace safety regulations (32–34), which is reflected in its alarming safety statistics. The severity of the construction safety problem in India highlights the urgent need to understand accident patterns in the sector. However, the lack of comprehensive accident databases hinders the identification of specific incident patterns, thereby limiting the development of targeted fall-prevention strategies. According to (71), falls are predicted to account for 50% of all construction accidents in India. Learning from past accidents is crucial to preventing similar hazardous situations from recurring (35). Therefore, this study aims to address this gap by analyzing publicly reported fatal falls in Indian construction using association rule mining (ARM). ARM is a powerful tool for discovering relationships and dependencies among variables within large datasets (36). In the present study, ARM was selected because the objective was exploratory: to identify recurring co-occurrence patterns among multiple categorical accident attributes. While several analytical approaches are available for accident analysis (15, 37), their suitability varies with the dataset’s nature and the purpose of the investigation. In data-constrained contexts, approaches that focus primarily on summarization, prediction, or predefined relationships may be less suitable for uncovering recurring interrelationships among multiple categorical variables. In contrast, ARM enables the extraction of transparent and interpretable association rules, making it particularly well-suited to identifying recurring combinations of worker characteristics, accident circumstances, and causal factors (38–40). The primary objective of this study was to investigate fatal falls in India and identify patterns and causal factors in the construction industry. By employing the ARM technique, this study seeks to uncover hidden patterns and relationships within datasets, providing valuable insights to inform decision-makers’ fall-prevention strategies.

## 2 Literature review

### 2.1 Fall accident analysis in construction

Falls are a leading cause of fatalities and injuries in the construction industry, accounting for approximately 33% of all construction-related fatalities (16). Understanding the causes and contributing factors of fall accidents is crucial to developing effective prevention strategies. The study of fall accidents has evolved over time, with various theoretical frameworks being developed to analyze and prevent these incidents. These frameworks include sequential causal models like Heinrich’s “domino theory,” epidemiological models such as Reason’s “Swiss cheese” model, and systemic models, such as AcciMap, STAMP (Systems Theoretic Accident Model and Processes), and FRAM (Functional Resonance Accident Model) (41–43). As a primary causal factor, human factors contribute to varying degrees of injury in accidents, underscoring their dominance in the causation of construction accidents (44, 45). Therefore, several studies have adopted the Human Factors Analysis and Classification System (HFACS) framework to investigate accident causes in the construction industry, highlighting the significant role of human factors and systemic problems (7, 46). The literature has extensively examined the variables contributing to the high rate of falls in construction, including victim demographics, occupational role, work or project type, and fall location (4, 17, 47). Researchers then used these variables to detect patterns through descriptive statistics and inferential models. A recurring theme is that fall risk is not determined by a single attribute; rather, certain outcomes are associated with combinations of variables, such as specific fall locations, worker role, and regional/site context (6). In particular, investigations that include causal-factor categories—such as the availability and effectiveness of fall protection or misuse/non-use of protection systems—often show that causal variables interact strongly with location and occupational variables that reflect experience and familiarity with safety procedures (17). Data-mining approaches strengthen this line of research by identifying frequent and “strong” association rules that link antecedent conditions to consequential causal factors (48, 49).

Prevention strategies, such as safety training, equipment, and protocols, have been developed to reduce the risk of falls (8). Many studies recommend targeted prevention based on identified combinations of high-risk variables rather than generic training. For example, when accident-variable patterns indicate repeated associations between scaffold-related work and non-use of fall protection, safety interventions are proposed at the task and location levels to ensure the availability of fall protection and strengthen supervision for high-risk activities (50, 51). Despite efforts to prevent falls in various regions, India has not seen similar initiatives. The lack of proper accident statistics in India, particularly in the construction sector, hinders the development of effective prevention strategies. This makes it challenging to understand the scope and causes of accidents, let alone develop targeted prevention strategies. In India, the scarcity of research on construction accidents, including falls, is a significant concern. While some studies have analyzed accidents using police records and other sources, these studies are limited, and it is unclear whether they accurately represent the magnitude of the problem (52, 71). Moreover, the lack of comprehensive data on fall accidents in India makes it difficult to identify patterns, including who is most affected, where they occur, and the causal factors behind these incidents. This study aims to address this knowledge gap by investigating patterns of fatal fall accidents in the Indian construction sector.

### 2.2 Data mining for accident analysis

As stated in the introduction, data mining techniques allow for the examination of vast amounts of data. According to Martínez-Rojas et al. (53), these techniques can reorganize data into a more interpretable format. Given the desired objective, various approaches can be employed, including grouping similar data points (e.g., clustering), uncovering relationships between variables [e.g., association rule mining (ARM)], and identifying unusual patterns (e.g., anomaly detection) (54). Among these, ARM was considered the most suitable technique in this study for analyzing large datasets on occupational accidents to uncover underlying patterns and trends. The application of ARM has been demonstrated in various accident-related studies, including casualty traffic crashes (55), maritime accidents (56), road accidents (57), and the analysis of ship traffic accidents (72) to uncover causal factors and interrelationships among factors. For construction accident analysis, the prevailing application of ARM has been to investigate and identify the underlying causal factors of accidents. For example, Cheng et al. (58) identified cause-and-effect relationships among accident factors in Taiwan’s construction industry, highlighting worker and management negligence as major contributors. Similar studies have been conducted in Korea (40) and Spain (39). Some studies have proposed accident causation models using ARM, which have developed and validated these models for various accident types, including construction collapses and falls, across regions such as Korea, Taiwan, China, and Turkey (38, 54, 59, 60). However, these studies have limitations in their analysis of specific accident types. These studies have emphasized the importance of exploring interrelationships among accident causal factors. Since then, ARM has been highlighted as a valuable approach for accident analysis, providing more intuitive results than traditional statistical methods. This is particularly useful given the complexity and multitude of factors involved. Although ARM is often associated with very large datasets, prior accident research has shown that it can also be meaningfully applied to domain-specific datasets when the purpose is exploratory pattern discovery among categorical variables (54). In such contexts, the value of ARM lies in identifying recurring combinations of factors that may otherwise remain hidden in conventional tabulations. This is especially relevant in construction safety research in data-constrained settings, where official databases may be unavailable or incomplete. Nevertheless, using ARM with smaller datasets requires careful threshold selection and cautious interpretation, because less frequent patterns may be unstable and the generated rules may be sensitive to the chosen support and confidence thresholds. Accordingly, in the present study, ARM was used as an exploratory analytical tool to identify recurring relationships within the available fatal fall cases. Given the complexity of the factors contributing to fatal falls in Indian construction, ARM could be a valuable tool for further analysis and for uncovering interrelationships among these factors.

## 3 Methodology

**Figure 1:**
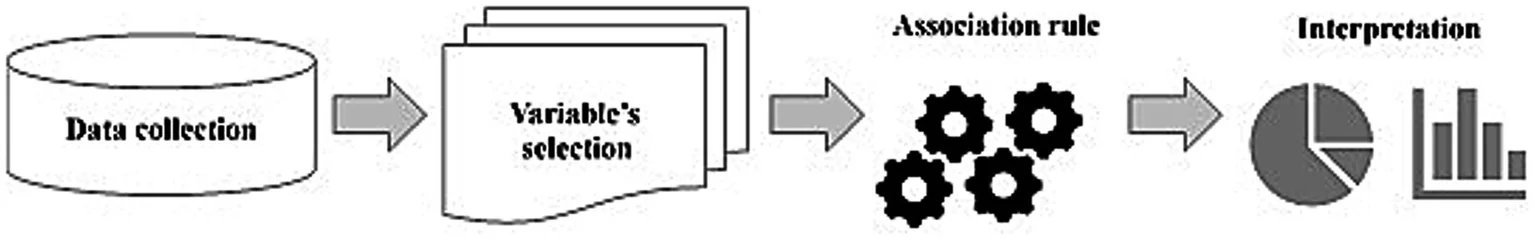
Research methodology framework.

The methodology employs a four-stage approach: data collection, variable selection, application of association rules, and interpretation of the results. Figure 1 illustrates the research methodology framework used in this study.

### 3.1 Data collection

**Figure 2:**
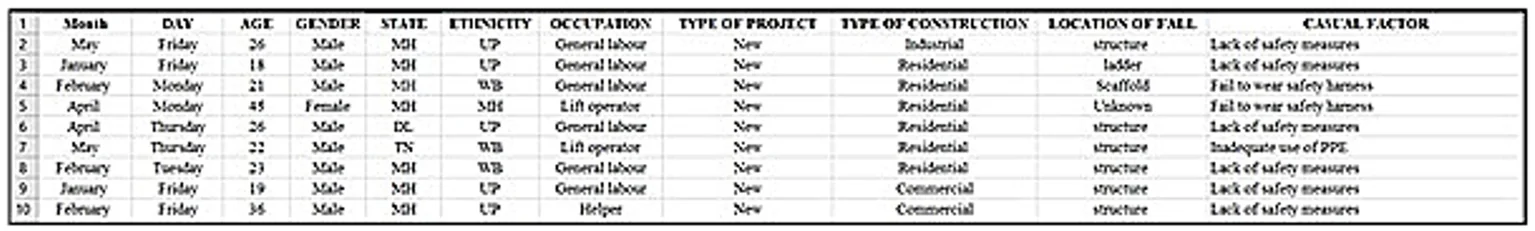
Sample data retrieved from the news articles.

The lack of a comprehensive, centralized database for construction accidents in India presented a significant challenge for this research. The National Crime Records Bureau (NCRB), responsible for collecting data on accidental deaths and suicides, does not track construction accidents specifically. A formal request for data on construction accidents since 2000, submitted under India’s Right to Information (RTI) Act to the Ministry of Home Affairs (MoHA), was unsuccessful; the researchers were informed that such records were not maintained. To overcome this substantial data gap, the researchers adopted an approach recommended in previous studies (13, 61) by using publicly available information from the digital archives of three major Indian newspapers: The Times of India, The Indian Express, and The Hindustan Times. These outlets were selected based on their high ranking in the SCImago media ranking (62), indicating their broad reach and likely comprehensive coverage of significant news events. A systematic search used relevant keywords, including “construction,” “fall accidents,” and “falls from height,” identifying a large initial dataset of 6,513 articles across the three news sources. While the initial search yielded many articles, the researchers found inconsistencies in the reporting of accident data across years. After a thorough random sampling and cross-checking of articles across various years, it was determined that consistently reliable data on construction fall accidents was only available from 2019 onwards. This limitation necessitated refining the dataset’s temporal scope. To focus the analysis and ensure data quality, the decision was made to concentrate specifically on fatal falls, recognizing that this approach would likely yield reliable data. The screening process, therefore, focused on articles published between January 1, 2019, and March 31, 2025. This resulted in a refined dataset of 351 articles. Further refinement was necessary to ensure data completeness and consistency. Articles lacking essential details—specifically, those failing to provide information on the victim’s age, occupation, fall location, and type of project—were excluded from the final analysis. This decision was based on prior research (13, 16–18, 23, 61), highlighting the importance of these variables in understanding accident patterns. This rigorous filtering process yielded a dataset of 126 cases. To enhance data reliability, each case was independently verified by comparing information from at least two other news articles reporting on the same incident. The data were then extracted through content analysis of news articles and systematically recorded in an Excel spreadsheet. The content analysis was conducted manually by a research team consisting of three members, who carefully read and reread each news article to extract relevant information. This process involved identifying and coding specific details, such as the date of the accident, the day of the accident, the victim’s age and gender, the state/region where the accident happened, the victims’ state of origin (SO), the victim’s occupation, the type of project and construction, the location of the accident, and the causal factors contributing to the falls. Figure 2 shows an example of raw data retrieved from the news articles. The selection of these variables was informed by prior research on construction accidents, which has consistently shown that they are among the most critical factors contributing to accidents (14, 39, 73). Each article was thoroughly examined, and the extracted information was manually entered into an Excel spreadsheet. The team members verified the accuracy of the entered data by rechecking the article and data entry using a triangulation approach. Specifically, each team member reviewed and verified the data with the other two members, forming a triangular verification process. This ensured that the information was accurate and reliable, and helped to minimize errors or discrepancies in the data.

### 3.2 Selection of variables

The next step was to select the variables and classify them based on the retrieved data. In this process, the variables were categorized into four distinct groups: individual (age, gender, workers’ SO, and occupation); project (project type and construction type); accident (month and day of the accident, state/region, and location of the fall); and causal factor. Certain variables, such as the month and day of the accident, project type, and worker gender, were excluded from the analysis due to data availability and relevance limitations. Specifically, data on the month and day of the accident, as well as project type, were not typically captured in accident reporting mechanisms, making it difficult to incorporate these variables into the study. Given the limited variability in certain variables (e.g., gender), they were excluded to ensure a robust analysis. The male-dominated construction industry’s characteristics justify focusing on male workers’ experiences, providing valuable insights into industry safety challenges. Despite these exclusions, other relevant variables were identified and included in the study to provide a comprehensive analysis. Following the selection of variables, the next step was to categorize them into meaningful groups to facilitate analysis and interpretation. Except for age and causal factors, all other variables were already clearly categorized through coding during retrieval. Therefore, the age was further categorized into categorical data (see Table 1), and then the causal factors were categorized based on four groups supported by the literature (15): fall protection existed but was wrongly used, referring to incorrect usage; no fall protection, indicating a lack of safety equipment; fall protection existed but was ineffective in use, where equipment failed to prevent falls; and fall protection existed but was not used, highlighting instances where available safety equipment was not worn or utilized by workers. The identified factors were then grouped into these four categories. For example, “lack of safety measures or inadequate use of PPE,” which was coded as “no fall protection.” Similarly, the factor “fail to wear safety harness” and related factors were grouped into “fall protection existed but was not used.” By grouping related causal factors, the researchers identified broader patterns and trends in the data. The description of accident variables and their categorizations are listed in Table 1.

**Table 1:** Accident variables and their categorization.

Description of accident variables	Categorization
Age of the victim involved in a fall.	18–20 (A1); 21–25 (A2); 26–30 (A3); 31–35 (A4); 36–40 (A5); >40 (A6)
Workers’ state of origin: The ethnic background of the victim involved in the fall.	UP (Uttar Pradesh) (M1), BH (Bihar) (M2), MH (Maharashtra) (M3), WB (West Bengal) (M4), AP (Andhra Pradesh) (M5), HR (Haryana) (M6), DL (Delhi) (M7), TN (Tamil Nadu) (M8), OR (Odisha) (M9), AS (Assam) (M19), MP (Madhya Pradesh) (M11).
Occupation: The job or role of the victim involved in the fall.	General labor (O1), helper (O2), lift operator (O3)
Type of construction: The specific type of construction work being performed.	Industrial (T1), residential (T2), commercial (T3)
Location of fall: The specific location where the fall occurred.	Scaffold (L1), structure (L2), ladder (L3), lift opening (L4)
State/region: The geographical state or region where the fall occurred.	UP (Uttar Pradesh) (S1), MH (Maharashtra) (S2), HR (Haryana) (S3), DL (Delhi) (S4), TN (Tamil Nadu) (S5), WB (West Bengal) (S6), GJ (Gujarat) (S7), RJ (Rajasthan) (S8)
Causal factor: The underlying reason or cause that contributed to the fall.	Fall protection existed but was wrongly used (C1), no fall protection (C2), fall protection existed but was ineffective in use (C3), fall protection existed but was not used (C4).

### 3.3 Association rule mining

ARM is a machine learning approach that automatically identifies significant associations between variables in large datasets. It detects co-occurrences of elements or attributes within events, enabling the discovery of interesting relationships and patterns that can inform decision-making (63). This study utilized the Apriori Algorithm to extract meaningful patterns from the data. The Apriori Algorithm is a popular, widely used method in ARM that automatically identifies significant associations among variables in large datasets (38). The algorithm works by identifying frequent item sets in the database and generating association rules from them. The association rules are typically expressed in a simple, intuitive format: X ⇒ Y, which reads “if X, then Y.” In this notation, X is the antecedent, or the “if” part, and Y is the consequent, or the “then” part. This means that the presence of X is expected to lead to Y. For example, if an association rule states “no fall protection ⇒ scaffold,” it implies that no fall protection (X) is likely to lead to a fall in scaffold (Y). This algorithm involves two key steps: identifying frequent item sets in the database and generating association rules from them. The algorithm uses three key metrics to evaluate the effectiveness and significance of each association rule: support, confidence, and lift.

Support measures the frequency with which X and Y co-occur in the database. It represents the proportion of transactions that contain both X and Y. It is calculated using the formula:


$$\text{Support}(X\cup Y)=\frac{\mathrm{num}(X\cup Y)}{\mathrm{num}(I)}=\frac{P(X\cup Y)}{P(I)}$$

where *P(X* ∪ *Y)* represents the probability of occurrence of the item set *X* ∪ *Y*, *P(I)* represents the probability of occurrence of the entire database I, *P(I)* = 1, *num(X* ∪ *Y)* represents the number of occurrences of the item set *X* ∪ *Y*, and *num(I)* represents the total number of occurrences in the database I.

Confidence measures the conditional probability that a transaction containing X also contains Y. It represents the percentage of transactions that include X and Y, given that they already contain X. The confidence is calculated using the following formula:


$$\text{Confidence}(X\to Y)=\frac{\text{Support}(X\cup Y)}{\text{Support}(X)}=\frac{P(X\cup Y)}{P(X)}$$

where *P(X)* represents the occurrence probability of item X and also corresponds to the support of item *X*, denoted as *Support(X)*.

The lift of an association rule is evaluated by comparing its confidence to the overall occurrence of item Y in the database. This is because a high confidence value may be influenced by the inherent high frequency of item Y. To account for this, lift is calculated as the ratio of the confidence to the independent occurrence of item Y, providing a more nuanced measure of the rule’s strength. The lift is computed using the following formula:


$$\text{Lift}(X\to Y)=\frac{\text{Confidence}(X\to Y)}{\text{Support}(Y)}=\frac{P(Y|X)}{P(Y)}$$

where the occurrence probability of item *Y* is denoted as *P(Y)*, the conditional probability of item *Y* given item *X*, denoted as *P(Y|X)*, and the support of item *Y*, denoted as *Support(Y)*, which provide the necessary context to evaluate the strength of the association rule. Effective strong association rules are identified by meeting the minimum support and confidence thresholds and exhibiting a lift greater than 1, indicating a meaningful relationship between X and Y (38, 59, 60).

The Apriori algorithm was implemented in Python 3.11 using the MLxtend library. To prepare the data for analysis, a one-hot encoding scheme was used to convert categorical variables into numerical form that the algorithm can process. This involved encoding each variable as a binary vector, where 1 indicates the presence of a particular category and 0 its absence. In ARM, thresholds play a crucial role in determining the quality and relevance of the extracted rules. The three primary thresholds used are support, confidence, and lift, which can be adjusted based on the dataset’s specific characteristics and desired mining results, allowing users to customize the mining process and extract meaningful insights (40). In this study, the Apriori algorithm was applied by iteratively adjusting and evaluating threshold values to balance rule stability, interpretability, and practical relevance. Because the dataset comprised 126 fatal fall cases, excessively high thresholds excluded potentially meaningful but less frequent accident patterns, whereas excessively low thresholds produced numerous weak or redundant rules, reducing interpretability. After repeated testing, the minimum support threshold was set to 0.05 (5%), meaning that a rule had to appear in at least approximately six cases to be considered, and the minimum confidence threshold was set to 0.50 (50%), indicating that the consequent had to occur in at least half of the cases containing the antecedent. These values are broadly consistent with prior ARM-based accident studies, which commonly adopt moderate threshold values and then refine the final rule set using lift. Given the exploratory purpose of the study, the threshold selection was not intended to maximize the number of rules, but rather to retain rules that were sufficiently recurrent and practically interpretable within a small but information-rich dataset. Using these thresholds, 226 initial association rules were generated. The results of the association rules were saved to CSV files for further analysis and interpretation. This enabled a detailed examination of relationships among variables and the identification of patterns and trends in the data.

## 4 Results

### 4.1 Dataset overview

The analysis of victim characteristics and accident trends revealed notable insights. The victims were predominantly young, with 37.30% in the 21–25 age group, and were largely from Uttar Pradesh, Bihar, and Maharashtra, accounting for 40.48, 16.67, and 16.67% of the sample, respectively. General laborers comprised 61.90% of victims, while residential buildings accounted for 76.19% of the project’s end use. The most common fall locations were from or with the structure (38.89%) and scaffolds (33.34%), with no fall protection used in 49.05% of cases. Additionally, falls through lift openings and ladders were less frequent, occurring in 13.49 and 3.17% of cases, respectively. Regional disparities in fatal fall accidents were also observed, with Maharashtra, Haryana, and Delhi reporting the highest number of fatal fall incidents.

### 4.2 Association rule analysis

**Figure 3:**
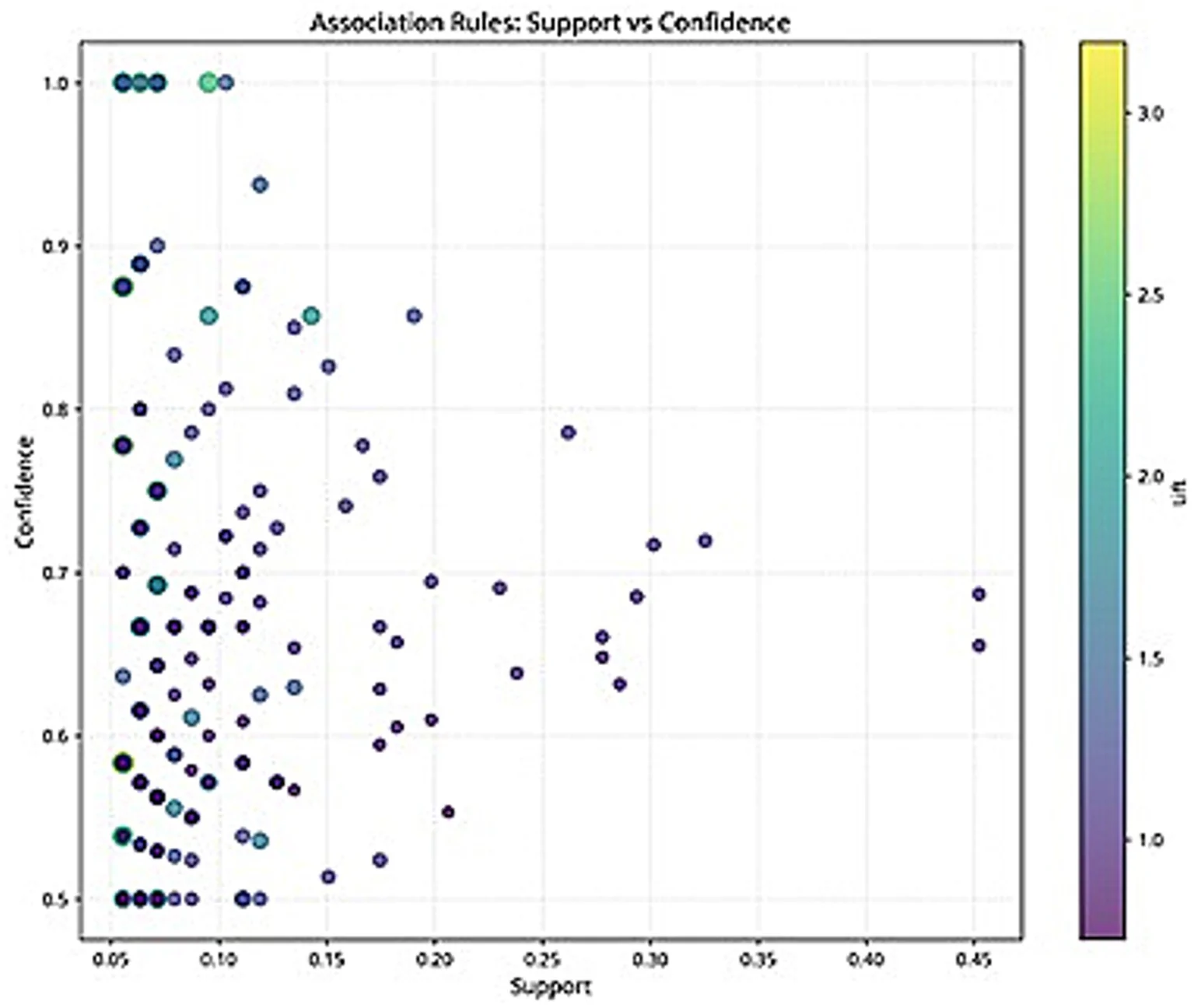
Scatter plot of all association rules, where each point represents a rule.

A scatter plot was created to visualize the relationship between support and confidence for all generated association rules (Figure 3). The plot displays support on the x-axis, confidence on the y-axis, and the points’ color and size represent the lift value. This visualization provides a comprehensive view of the rules’ characteristics, enabling the identification of trade-offs and patterns in the distribution of support, confidence, and lift across all rules. Notably, rules with high lift values, even if they have moderate support and confidence, are of particular interest, as they indicate strong deviations from expected co-occurrences and highlight potentially valuable insights.

Strong association rules are identified by meeting specific criteria: a lift greater than 1, indicating a positive association between the antecedent and consequent variables. Upon examining the obtained association rules, it was observed that some had lift values less than 1. To strengthen the rules, a higher lift threshold of 1.1 was set, a value commonly recommended in the literature (54, 59, 60). By applying this refined lift threshold, 148 strong association rules were identified, leaving 78 rules that did not meet the threshold and thus indicated weaker associations between the variables. These strong association rules provide valuable insights into the relationships among the variables in the dataset. Furthermore, the large number of obtained rules (148) and the presence of redundant information necessitate a more organized approach to analysis. To facilitate a more refined and structured examination of the association rules, the 148 rules were categorized into six consequent types: causal factors, workers’ SO, location of fall, age, occupation, and state/region. Notably, to facilitate a more cohesive analysis, association rules with identical antecedents are aggregated and examined collectively, while rules with unique or disparate antecedents are treated as standalone rules. The distribution of rules across the categories was identified as: 33 for causal factors, 29 for workers’ SO, 19 for location of fall, 6 for age, 31 for occupation, and 30 for state/region. The results for the top 10 rules in each category (except age) are presented in Tables 2–6. Notably, because only a limited number of rules were generated for the age category, all 6 association rules are presented in Table 7.

**Table 2:** Significant association rules based on causal factors.

Antecedents					Consequents	Support	Confidence	Lift
M1		A1			C2	0.071	1.000	1.45
M2				L1	C2	0.071	1.000	1.45
M9					C2	0.071	1.000	1.45
M2			S2		C2	0.071	1.000	1.45
M9	O1			L1	C2	0.063	1.000	1.45
M2	O1		S2		C2	0.056	1.000	1.45
		A6			C2	0.056	1.000	1.45
	O1	A5			C2	0.071	0.900	1.30
		A4		L2	C2	0.063	0.889	1.29
			S4	L1	C2	0.063	0.889	1.29

**Table 3:** Significant association rules based on a worker’s state of origin.

Antecedents					Consequents	Support	Confidence	Lift
S4			O1		M2	0.056	0.538	2.51
S3		C2	O1		M1	0.056	1.000	2.38
S3		C2			M1	0.071	1.000	2.38
S3			O1		M1	0.063	1.000	2.38
S3					M1	0.095	1.000	2.38
S4					M2	0.071	0.500	2.33
	A1	C2			M1	0.071	0.692	1.65
	A1				M1	0.071	0.692	1.65
S2	A2	C2			M1	0.063	0.667	1.58
	A2	C2	O1		M1	0.079	0.588	1.40

**Table 4:** Significant association rules based on the location of a fall.

Antecedents					Consequents	Support	Confidence	Lift
C2	S4	O1			L1	0.056	0.875	2.63
C1		O1			L2	0.056	1.000	2.33
C2	S4				L1	0.063	0.667	2.00
	S4	O1			L1	0.063	0.615	1.85
C2	S2				L2	0.056	0.778	1.81
C2					L2	0.063	0.667	1.56
	S4				L1	0.071	0.500	1.50
C1			A4		L2	0.063	0.615	1.44
		O1	A3		L2	0.111	0.583	1.36
			A3		L2	0.127	0.571	1.33

**Table 5:** Significant association rules by occupation.

Antecedents					Consequents	Support	Confidence	Lift
S2		A3	C2		O1	0.071	1.000	1.52
	M3	A3			O1	0.056	1.000	1.52
S2		A3		L2	O1	0.056	1.000	1.52
S2		A3			O1	0.119	0.938	1.42
			C2	L4	O1	0.063	0.889	1.35
	M2		C2	L1	O1	0.063	0.889	1.35
	M2			L1	O1	0.063	0.889	1.35
S4				L1	O1	0.063	0.889	1.35
	M2	A3		L2	O1	0.056	0.875	1.33
S4			C2	L1	O1	0.056	0.875	1.33

**Table 6:** Significant association rules based on state/region.

Antecedents					Consequents	Support	Confidence	Lift
C1				L2	S2	0.056	0.875	1.93
		O1	M3		S2	0.095	0.857	1.89
			M3		S2	0.143	0.857	1.89
C4		O1			S2	0.079	0.769	1.70
C1					S2	0.071	0.750	1.66
C2			M3		S2	0.071	0.750	1.66
	A5				S2	0.079	0.667	1.47
C4					S2	0.095	0.667	1.47
C4	A3	O1			S2	0.071	0.643	1.42
L4		O1			S2	0.056	0.636	1.41

**Table 7:** Significant association rules by age group.

Antecedents					Consequents	Support	Confidence	Lift
M3			O1		A3	0.056	0.500	2.25
M1	S2	C2			A2	0.063	0.727	1.95
		C4	O1		A2	0.063	0.615	1.65
		C4			A2	0.087	0.611	1.64
M1	S2		O1		A2	0.063	0.571	1.53
M1	S2				A2	0.087	0.550	1.47

According to Table 2, “no fall protection” (C2) consistently emerges as the dominant consequent across multiple antecedent combinations. This indicates that the absence of fall protection is the most critical causal factor associated with fatal falls. Several ethnic workers’ SO groups, particularly workers from Uttar Pradesh (M1), Bihar (M2), and Odisha (M9), show strong associations with accidents in which fall protection was absent. For example, the rule linking M1 → C2 has a confidence of 1.000 and a lift of 1.45, meaning that every accident involving workers from Uttar Pradesh in this subset was associated with the absence of fall protection. Similarly, location-specific antecedents such as scaffold (L1) and state-level antecedents like Maharashtra (S2) also strongly co-occur with the absence of fall protection. The rule M2 → L1 → C2 underscores that scaffold-related falls among workers from Bihar almost invariably lacked fall protection. Occupational antecedents further reinforce this pattern: general laborers (O1) combined with workers’ SO groups (M2, M9) or specific states (S2) consistently point to “no fall protection” as the consequent, again with perfect confidence values (1.000) and elevated lift scores (1.45). Age-related antecedents also appear in the causal factor rules. For instance, victims aged >40 (A6) and those in the 36–40 range (A5) are disproportionately linked to accidents without fall protection. The rule O1 + A5 → C2 shows a confidence of 0.900 and a lift of 1.30, suggesting that older general laborers are particularly vulnerable when fall protection is absent. Likewise, structural locations such as L2 (structure) and regional antecedents like Delhi (S4) also show elevated associations with no fall protection, though with slightly lower confidence (0.889) and lift (1.29).

Table 3 elucidates the strong association between workers’ SO and patterns of fall accidents. Individuals from Uttar Pradesh (M1) and Bihar (M2) are disproportionately represented among accident victims. For example, rules such as S3 → C2 → M1 and S3 → O1 → M1 show perfect confidence values (1.000) and high lift scores (2.38), meaning that whenever accidents occurred in Haryana (S3) with causal factors like no fall protection (C2) or occupations such as general labor (O1), they were consistently linked to victims from Uttar Pradesh. Lift values above 2.0 indicate that these associations are more than twice as likely as expected at baseline, underscoring disproportionate vulnerability in this group. Similarly, M2 (Bihar) emerges as another workers’ SO category at elevated risk of accidents. The rule S4 → O1 → M2 shows a lift of 2.51, indicating that general laborers from Bihar working in Delhi (S4) are more than 2.5 times as likely to be involved in fall accidents. Although the confidence here is moderate (0.538), the high lift suggests a high relative risk. Other rules, such as S4 → M2, reinforce this pattern, pointing to Delhi as a critical hotspot for Bihar-origin workers. Age also interacts with workers’ SO in meaningful ways. For instance, A1 (18–20 years) → C2 → M1 shows that younger workers from Uttar Pradesh are disproportionately linked to accidents without fall protection. This suggests that younger migrant workers may face compounded risks. Similarly, rules involving A2 (21–25 years) combined with causal factors and occupations again point to M1, though with slightly lower confidence (0.588–0.667) and lift (1.40–1.58).

Table 4 highlights strong links between fall location categories and both causal factors and vulnerable worker groups. The most striking pattern is the repeated linkage between scaffold-related falls (L1) and the absence or misuse of fall protection. For example, the rule C2 + S4 + O1 → L1 shows a high lift of 2.63, meaning that general laborers in Delhi (S4) working without fall protection are more than two and a half times more likely to experience scaffold-related fall accidents. Falls in structure (L2) also emerge prominently, particularly when combined with causal factors such as C1 (fall protection existed but was wrongly used). The rule C1 + O1 → L2 demonstrates perfect confidence (1.000) and a lift of 2.33, indicating that when general laborers were involved in accidents involving improperly used fall protection, the fall almost always occurred from a structure. Other rules reinforce these patterns with slightly lower confidence but still elevated lift values. For instance, C2 + S2 → L2 (confidence 0.778, lift 1.81) indicates that falls in Maharashtra (S2) without fall protection are disproportionately linked to structures. Similarly, C2 → L2 (confidence 0.667, lift 1.56) shows that across the dataset, missing fall protection consistently increases the likelihood of structural falls. Age-related antecedents also appear in this category. Workers aged 31–35 (A4) and 26–30 (A3) are repeatedly associated with structural falls. For example, O1 + A3 → L2 (confidence 0.583, lift 1.36) and A3 → L2 (confidence 0.571, lift 1.33) suggest that younger adult workers, particularly general laborers, are disproportionately exposed to structural fall risks.

Table 5 indicates that occupation is strongly associated with the risk of fatal falls when combined with state, age, workers’ SO, and causal factors. The most dominant pattern is the repeated linkage of O1 (general labor) with unsafe conditions in Maharashtra (S2) and among workers aged 26–30 (A3). For example, the rules S2 + A3 + C2 → O1 and S2 + A3 + L2 → O1 both show perfect confidence (1.000) and lift values of 1.52, meaning that whenever fatal fall accidents involved workers in Maharashtra aged 26–30, they almost always occurred among general laborers, with a 52% higher likelihood compared to baseline. This underscores the vulnerability of younger adult general laborers in high-risk states. Workers’ SO also plays a role in occupation-based associations. The rule M3 + A3 → O1 (confidence 1.000, lift 1.52) indicates that workers from Maharashtra (M3) aged 26–30 are consistently associated with general labor accidents. Similarly, M2 (Bihar) → C2 + L1 → O1 (confidence 0.889, lift 1.35) indicates that scaffold-related falls among Bihar-origin workers without fall protection disproportionately involve general laborers. These rules suggest that migrant workers from Bihar and Maharashtra are overrepresented in general labor accident scenarios, particularly in scaffold and structural work. Location-specific antecedents further reinforce this pattern. The rule S4 → L1 → O1 (confidence 0.889, lift 1.35) shows that scaffold-related accidents in Delhi (S4) are strongly associated with general laborers. Similarly, C2 + L4 → O1 (confidence 0.889, lift 1.35) highlights that lift-opening accidents without fall protection disproportionately involve general laborers. These findings suggest that general laborers are consistently exposed to high-risk environments across multiple states/regions. Age continues to be a significant factor. The rule M2 + A3 → L2 → O1 (confidence 0.875, lift 1.33) shows that Bihar-origin workers aged 26–30 are particularly vulnerable to structural falls as general laborers. Likewise, S2 + A3 → O1 (confidence 0.938, lift 1.42) reinforces that younger adult workers in Maharashtra are disproportionately represented in general labor accident cases.

Table 6 demonstrates that Maharashtra (S2) is consistently the most vulnerable region. Several rules demonstrate that falls occurring in Maharashtra are disproportionately linked to unsafe causal factors, workers’ SO, and occupational roles. For instance, the rule C1 → L2 → S2 (confidence 0.875, lift 1.93) indicates that when fall protection was present but misused, accidents almost always occurred from structures in Maharashtra. Workers SO further reinforces this pattern. The rules O1 + M3 → S2 (confidence 0.857, lift 1.89) and M3 → S2 (confidence 0.857, lift 1.89) highlight that worker from Maharashtra (M3), particularly general laborers, are disproportionately represented in accidents within the same state. The high support values (0.095–0.143) indicate that these associations are not isolated but recurring across the dataset. This suggests that local workers, as well as migrant laborers from Maharashtra, face systemic risks in construction environments. Causal factors such as C4 (fall protection was in place but not used) also appear prominently. The rule C4 + O1 → S2 (confidence 0.769, lift 1.70) indicates that general laborers in Maharashtra often fail to use available fall protection, increasing the risk of accidents. Similarly, C2 → M3 → S2 (confidence 0.750, lift 1.66) indicates that the absence of fall protection among Maharashtra-origin workers is a recurring problem. Age-related antecedents also interact with state-level risks. For example, A5 (36–40 years) → S2 (confidence 0.667, lift 1.47) and C4 + A3 + O1 → S2 (confidence 0.643, lift 1.42) show that mid-aged workers, particularly general laborers, are disproportionately involved in accidents in Maharashtra. Moreover, the rule L4 + O1 → S2 (confidence 0.636, lift 1.41) shows that lift-opening accidents in this state disproportionately involve general laborers.

Table 7 shows how age interacts with workers’ SO, occupation, and causal factors to shape patterns of fatal fall risk. The most consistent pattern is the vulnerability of workers aged 21–25 (A2), who appear in multiple rules with elevated confidence and lift values. For example, the rule M1 + S2 + C2 → A2 (confidence 0.727, lift 1.95) shows that workers from Uttar Pradesh (M1) in Maharashtra (S2), when exposed to accidents without fall protection, are disproportionately aged 21–25. Furthermore, the rules C4 + O1 → A2 (confidence 0.615, lift 1.65) and C4 → A2 (confidence 0.611, lift 1.64) indicate that when fall protection was present but not used, victims were often in the 21–25 age group. Similarly, M1 + S2 + O1 → A2 (confidence 0.571, lift 1.53) and M1 + S2 → A2 (confidence 0.550, lift 1.47) confirm that Uttar Pradesh-origin general laborers in Maharashtra are consistently overrepresented in accidents within this age bracket. The age group 26–30 (A3) also appears in the rules, though less frequently. For instance, M3 + O1 → A3 (confidence 0.500, lift 2.25) shows that general laborers from Maharashtra (M3) are disproportionately represented in accidents at this age. Despite the lower confidence, the high lift value (2.25) indicates that this association is more than twice as likely compared to baseline, suggesting a significant relative risk.

## 5 Discussion

This study employed association rule mining to uncover critical patterns and relationships in fatal falls in the Indian construction industry, building on the identification of 148 strong association rules. The findings provide a granular understanding of the interplay between causal factors, demographic vulnerabilities, occupational roles, and geographical contexts. The most salient finding of this study is the overwhelming association between fatal falls and the complete absence of fall protection measures. Across diverse combinations of antecedents, the absence of any fall protection system emerged as the primary causal factor. This pattern suggests that in the Indian context, fall prevention is frequently nonexistent on construction sites. This finding aligns with previous studies, indicating that the absence of fall protection equipment accounts for 30–70% of fall accidents in construction (15–17). In contrast, literature from developed economies, including the United States, South Korea, and European nations, typically identifies the improper use or ineffectiveness of fall protection as a significant contributor to fall incidents (16, 17). However, in India, there is a distinct deviation: the primary issue is not the misuse of equipment or equipment failure, but rather the systematic failure to provide basic safety infrastructure. This aligns with Bansal's (33) observation regarding the weak enforcement of workplace safety regulations in India. Unlike jurisdictions with robust occupational safety frameworks, such as OSHA standards in the US or Health and Safety Executive (HSE) guidelines in the UK, India lacks a comprehensive and enforceable regulatory mechanism for construction safety. While the Building and Other Construction Workers (BOCW) Act, 1996, Occupational Safety, Health and Working Conditions (OSH) Code of 2020, and the Bureau of Indian Standards have developed specific standards for workplace safety, including the IS 3521 series, which covers full-body harnesses, lanyards, self-retracting lifelines, and anchorage systems, these provisions exist legislatively, but implementation remains fragmented. Many contractors and small-scale firms disregard statutory safety requirements due to minimal inspection regimes and negligible penalties for non-compliance. Furthermore, the near-perfect confidence values linking specific workers’ SO groups to the absence of fall protection highlight a critical dimension of social vulnerability. Migrant workers from economically disadvantaged states—particularly Uttar Pradesh, Bihar, and Odisha—are disproportionately exposed to worksites lacking basic safety infrastructure (64). These individuals typically occupy the lowest tiers of the construction hierarchy, employed as casual daily-wage laborers without formal contracts or safety training (65, 66). Their transient nature and lack of bargaining power render them highly susceptible to exploitation by contractors who prioritize cost reduction over worker safety. While Shao et al. (14) have documented elevated fall risks among vulnerable worker populations globally, the Indian context is further exacerbated by the extreme informality of the labor market, in which an estimated 87% of construction workers operate in the unorganized sector without social security coverage (67, 68).

The analysis identified scaffolds and building structures as the most frequent locations for fatal falls, with both strongly associated with the absence of fall protection. Scaffold-related accidents, in particular, showed strong associations with specific worker profiles; for instance, general laborers from Bihar and workers in Delhi who lacked fall protection were highly likely to experience scaffold-related incidents. This finding corroborates international literature, which has long recognized scaffolds as high-risk environments (4, 8). However, the Indian context introduces unique variables to this pattern. Scaffolding in India is frequently constructed from bamboo, wood, or poorly maintained metal pipes rather than from engineered systems compliant with international standards. Although bamboo and wood scaffolding are cost-effective, they lack the structural integrity and safety features—such as guardrails, mid-rails, and toe boards—found in standardized systems. Moreover, the erection and dismantling of scaffolds are typically performed by workers without specialized training, resulting in unstable platforms and unprotected edges. The data indicate that scaffold falls frequently occur in the total absence of fall protection, suggesting that basic measures such as safety nets, guardrails, or personal fall arrest systems are rarely used. Structural falls—defined as falls from building edges, roofs, or unfinished floors—also emerged as a prominent cause, particularly when fall protection was present but misapplied. This distinction is significant: unlike scaffold falls, where protection is often entirely absent, structural falls sometimes involve the improper use of equipment. This may reflect a deficiency in training on proper harness anchorage, inadequate supervision, or the use of damaged or incompatible equipment. While López-Arquillos et al. (28) noted that improper use of fall protection is a global issue, in India, this is compounded by the near-total absence of formal safety training programs and the prevalence of untrained site supervisors.

Age-stratified analysis revealed that workers aged 18–25 years constitute a disproportionately high-risk group, with strong associations linking this demographic to the absence of fall protection, migrant status from Uttar Pradesh, and employment in Maharashtra. Workers aged 26–30 also appeared frequently in association rules, particularly general laborers from Maharashtra. This pattern is consistent with studies by Al-Bayati and York (15) and Shao et al. (14), which have identified young workers as overrepresented among those involved in fall incidents, attributing this to inexperience, risk-taking behavior, and inadequate safety mentorship. However, the Indian construction industry presents additional risk amplifiers. Young workers from impoverished rural backgrounds often enter the construction industry as their first formal employment, having received no prior safety training (69). The lack of structured onboarding or apprenticeship programs means these workers are assigned hazardous tasks—such as working at heights on scaffolds or at structural edges—without being shown safe practices. Furthermore, the prevalent piece-rate payment system incentivizes speed over safety; young workers, eager to demonstrate productivity, often bypass available safety measures. Notably, workers aged 36–40 also exhibited elevated associations with the absence of fall protection, suggesting a bimodal risk distribution: younger workers lack experience and safety awareness, while older workers may suffer from reduced physical capabilities, slower reaction times, or a fatalistic attitude developed over years of exposure to unsafe work environments. While Nykänen et al. (29) recommended targeted safety training for young workers, the Indian context necessitates differentiated interventions that address both extremes of age.

Geographic analysis identified Maharashtra, Delhi, and Haryana as the regions with the highest concentration of fatal fall incidents, each exhibiting distinct patterns. Maharashtra emerged as the most consistently vulnerable region, with strong associations linking the state to the improper use of fall protection, structural falls, and general labor occupations. Delhi demonstrated strong associations with scaffold-related falls and workers originating from Bihar, while Haryana showed near-perfect associations with workers from Uttar Pradesh. These regional patterns can be attributed to several interconnected factors. Maharashtra, particularly the Mumbai metropolitan region, hosts one of the highest concentrations of high-rise construction activity in India. The density of tall building projects creates frequent opportunities for falls from height, and the pressure to complete projects rapidly in a competitive real estate market often leads to compromised safety standards. The finding that fall protection existed but was misused or ignored in Maharashtra suggests that safety equipment may be present nominally—to satisfy regulatory requirements for large projects—but is inadequately deployed or disregarded in practice. Delhi and its surrounding National Capital Region (NCR), including Haryana, have experienced rapid urbanization and construction booms, attracting massive inflows of migrant labor from Bihar and Uttar Pradesh. The strong association between Delhi, scaffold falls, and Bihar-origin workers likely reflects the concentration of migrant labor in specific construction niches. Migrant workers from Bihar are often channeled into scaffold erection and formwork activities through informal labor contractors who recruit along regional networks. These contractors, operating with minimal oversight, frequently prioritize cost savings over safety, providing neither fall protection equipment nor training. The perfect confidence values linking Haryana-to-Uttar Pradesh workers reinforce this pattern of regional labor migration shaping accident profiles.

The consistent association of “general labor” with nearly every high-risk pattern—absence of fall protection, scaffold and structural falls, young age groups, and specific workers’ SO and regional backgrounds—underscores the precarious position of unskilled workers within the Indian construction hierarchy. General laborers in India perform a wide range of tasks at heights, including carrying materials on scaffolds, assisting masons on structural edges, and cleaning lift openings, yet they receive the least safety training and have the least access to personal protective equipment. This finding aligns with international research indicating that laborers are overrepresented in construction fatalities (17, 23). However, the situation in India is exacerbated by the absence of formal employment relationships. Most general laborers are hired on a daily-wage basis through labor contractors who bear no legal liability for workplace injuries. Unlike in jurisdictions where employers are legally mandated to provide fall protection training and equipment, Indian contractors can evade responsibility by classifying workers as “casual” or “self-employed.” The weak implementation of the Building and Other Construction Workers Welfare Cess Act, which mandates safety provisions and welfare benefits, means that even when funds are collected, they rarely translate into tangible on-site safety improvements.

### 5.1 Implications

This study carries significant theoretical, practical, and policy implications for improving fall safety in the Indian construction industry. From a theoretical perspective, the association rules reveal that failures occur systematically across regulatory, organizational, technical, and individual levels, with specific worker profiles experiencing compounded vulnerabilities based on the intersection of migrant status, age, occupation, and geographic context. This extends existing theoretical frameworks by showing that, in weakly regulated environments, accident causation follows predictable, patterned trajectories rather than random occurrences.

From a practical standpoint, the findings provide actionable guidance for construction contractors and managers. The overwhelming association between scaffold-related falls and the complete absence of fall protection suggests that scaffold safety should be the highest priority for immediate intervention. Contractors should implement mandatory pre-use scaffold inspections using standardized checklists, ensure complete guardrail systems are installed before any work commences, and provide personal fall arrest systems with proper anchorage points. Given that bamboo and wood scaffolding remain prevalent due to cost considerations, developing low-cost, standardized safety guidelines specifically for bamboo and wood scaffold construction—including requirements for guardrails, mid-rails, and toe boards—could significantly reduce risks in the small- and medium-enterprise sector, where engineered scaffolding is unaffordable. For general laborers, who emerged as the most vulnerable occupational group, employers should provide task-specific rather than generic safety training. The finding that young workers are disproportionately represented in fatal falls suggests the need for structured induction programs that include supervised work periods, peer mentoring, and realistic fall consequence demonstrations.

The policy implications of this study are equally substantial. The regional concentration of fall accidents in Maharashtra, Delhi, and Haryana indicates that enforcement resources should be strategically prioritized in these high-risk states. Regulators should conduct targeted inspection campaigns focused on scaffold safety and the availability of fall protection, with penalties for non-compliance that meaningfully exceed the cost of providing equipment—a condition that currently does not exist, as fines are sufficiently low that many contractors treat them as a routine cost of doing business rather than a deterrent. The finding that fall protection is entirely absent points to a fundamental regulatory failure. Policymakers should consider mandating that fall protection plans be submitted and approved before construction permits are issued for any project involving work at height, with random audits conducted during active construction, and the authority to issue stop-work orders for non-compliance. Additionally, the absence of a centralized accident database—highlighted by the researchers’ unsuccessful Right to Information request—should be addressed by mandating that all construction fatalities be reported to a national online portal within 24 h, with legal penalties for non-reporting.

For worker welfare organizations and trade unions, the disproportionate representation of young migrant workers from Uttar Pradesh and Bihar suggests that safety awareness campaigns should be conducted in source communities before workers migrate. Welfare organizations could establish help desks in destination cities such as Mumbai and Delhi, where workers can anonymously report unsafe conditions and receive information about their legal rights to safe working conditions. The insurance and financial sectors also have a role to play: insurance companies offering workers’ compensation policies could use the identified risk patterns to develop differentiated premium structures that reward contractors with documented fall-protection programs, while financial institutions financing construction projects could require evidence of fall-protection compliance as a condition for loan disbursement. Finally, educational institutions in India should prioritize fall-prevention construction into their curricula to consider safe means of access and fall protection during the design phase.

### 5.2 Limitations

This study has several limitations that should be acknowledged. First, the primary limitation is the reliance on online news articles to identify accident cases, which may lack comprehensive details or context. Newspapers often do not provide sufficient data, making it challenging to compare incidents across regions or periods. A standardized reporting form would help ensure consistent data collection and more accurate root cause identification. This limitation has also been highlighted in previous studies, including Al-Bayati and York (15) and Dong et al. (23). Additionally, the articles might omit critical factors such as the lack of safety measures, the safety training received by workers or provided by employers, project background information (such as project size and cost), and specific circumstances leading to the fall, which limit the depth of analysis. Moreover, the study focused on only three news outlets to retrieve accident cases from January 1, 2019, to March 31, 2025, which may restrict the breadth of information available for analysis. In future research, it is advisable to include a wider variety of news outlets to capture a broader range of accident cases. Expanding the data collection period would also allow researchers to analyze more incidents, leading to a better understanding of trends and patterns in fatal falls. Additionally, incorporating more local newspapers from different regions could uncover accident cases that may not be reported in national publications.

Second, a significant limitation concerns the inability to identify the size and type of construction firms represented in the dataset. The news articles used as data sources typically do not disclose whether the accident occurred at a site operated by a large, established construction company or a small or medium-sized enterprise. Consequently, it is impossible to determine whether the recorded cases disproportionately represent smaller firms, which may lack the resources and organizational capacity to implement fall protection measures, or whether larger firms are also represented but their accidents are reported differently. Large-scale construction companies in India are more likely to have formal safety management systems, dedicated safety personnel, and fall-protection equipment available on-site. It is also possible that the recorded data are primarily from medium and small firms, which may not accurately represent the safety performance of larger organizations. However, it must be emphasized that the primary aim of this study was not to compare safety performance across different types of organizations, but rather to identify accident patterns at the national level and to visualize the pervasive poor safety performance across the Indian construction industry.

Several important factors, such as project cost, the decedents’ construction experience, the type of activity, and the height of falls, were not covered in this study due to the limitations above. These factors are crucial for understanding where preventive measures should be focused. For instance, small to medium-sized construction projects, which often have lower project costs, tend to experience a higher incidence of falls (4, 17). This trend can be attributed to smaller companies frequently executing projects with poor safety records and inadequate safety protocols compared to larger firms (16). Additionally, workers’ experience level plays a significant role; less-experienced workers are more likely to be involved in accidents, particularly in environments where safety protocols are not strictly enforced. Furthermore, the type of activity at the time of an accident is crucial for identifying specific hazards and risk factors associated with different tasks. Finally, fall height is critical, as falls from greater heights generally result in more severe injuries or fatalities.

Lastly, the study focused exclusively on fatal falls, thereby excluding the broader spectrum of non-fatal injuries that contribute to the overall safety burden. Furthermore, newspapers are inherently selective in their coverage and are more likely to report fatal than non-fatal accidents; therefore, the patterns identified in this study may not fully represent non-fatal fall accidents, which may involve different causal factors, worker demographics, or locations. Reporting quality also varies across newspapers and regions, meaning accidents in remote areas or involving workers from marginalized communities may receive less coverage, leading to underrepresentation in the dataset. Because the dataset was derived from newspaper reports and included only 126 fatal cases, the results should be interpreted as exploratory and indicative of patterns rather than statistically representative of all fall accidents in the Indian construction industry. The identified rules highlight recurring combinations within the available dataset, but they should not be treated as population-level estimates. Nevertheless, in the absence of official national accident records, the dataset provides an important starting point for evidence generation in a severely under-documented context. Despite these limitations, the study successfully leverages available public data to uncover systemic patterns that have remained undocumented in the Indian context. The identified associations provide a robust foundation for hypothesis generation and the design of targeted, evidence-based safety interventions, serving as a critical first step toward improving construction safety in India.

## 6 Conclusion

Falls are the most serious type of accident in the construction industry. While global research has identified trends in fall incidents, a lack of focused studies in India has hindered the development of targeted preventive measures. This study set out to address a critical gap in construction safety research by investigating patterns of fatal fall accidents in the Indian construction industry, a context where comprehensive accident databases are notably absent. Using ARM applied to 126 fatal fall cases extracted from newspaper reports from January 1, 2019 to March 31, 2025, the study successfully identified meaningful patterns and interrelationships among causal factors, victim demographics, occupational roles, fall locations, and geographic contexts. The most salient finding is the overwhelming dominance of “no fall protection” as the causal factor in fatal falls, consistently across nearly all combinations of antecedents. This indicates that the fundamental problem in Indian construction is the absence of safety equipment. This finding aligns with earlier observations of weak regulatory enforcement in India but provides empirical specificity by quantifying the strength of associations between the absence of fall protection and specific worker profiles, locations, and regions. The study also revealed that young workers, general laborers, and migrant workers from Uttar Pradesh and Bihar face disproportionately high risks, with these vulnerabilities compounding in high-construction-intensity states such as Maharashtra, Delhi, and Haryana. Scaffold-related and structure-related falls were the most common fall locations and were both strongly associated with missing or improperly used fall protection. This study makes an important contribution as the first systematic analysis of fatal fall patterns in the Indian construction industry using data mining techniques. The findings provide an evidence-based foundation for targeting fall-prevention interventions to the most vulnerable workers, locations, and regions. Future research should aim to incorporate primary data collection methods—including site observations, worker surveys, and interviews with safety professionals—to validate and extend these findings. Finally, reducing the unacceptable toll of fall fatalities in Indian construction will require not only targeted interventions but also fundamental reforms in regulatory enforcement and the formalization of the industry’s vast informal workforce.

## Data Availability

The raw data supporting the conclusions of this article will be made available by the authors, without undue reservation.
